# Preschool Metacognitive Skill Assessment in Order to Promote Educational Sensitive Response From Mixed-Methods Approach: Complementarity of Data Analysis

**DOI:** 10.3389/fpsyg.2019.01298

**Published:** 2019-06-13

**Authors:** Elena Escolano-Pérez, Maria Luisa Herrero-Nivela, M. Teresa Anguera

**Affiliations:** ^1^Faculty of Education, University of Zaragoza, Zaragoza, Spain; ^2^Faculty of Psychology, Institute of Neurosciences, University of Barcelona, Barcelona, Spain

**Keywords:** mixed methods, systematic observation, preschoolers, metacognitive skills, T-pattern detection, lag sequential analysis, polar coordinate analysis, educational practice

## Abstract

A child's metacognitive skills contribute significantly to their learning and success. However, very few studies are focused on these skills at early education and most of them are carried out from inappropriate methodological perspectives for the characteristics of the youngest students. To overcome such limitations, it is essential to carry out observational studies that analyze children's metacognitive behaviors in the natural and habitual context of children's learning, as well as appropriate tasks for their level of development. The aim of this study was to analyze the sequential and associative structure of the metacognitive skills used by 5-year-old children throughout the resolution of a playful task (a puzzle). It was interesting to know if there were different hidden structures in the use of metacognitive skills in the children who solved the puzzle and those who did not. From the methodological approach, this work was located in the perspective of mixed methods which is characterized by the integration of qualitative and quantitative elements. This integration was carried out from the “connect” option. The integration involved developing quantitizing, as one of its possibilities. Recent scientific literature has considered systematic observation, in which the QUAL-QUAN-QUAL macro stages take place, as a mixed method itself. Consequently, systematic observation was applied, because it was suitable for our aim. A Nomothetic/Punctual/Multidimensional observational design was used. The playful activity of 44 preschool children solving the puzzle individually was coded. It allowed us to obtain data matrices that respond to the QUAL stage. Regarding the QUAN stage, once the quality of data was controlled, the records were further analyzed by differentiating two groups of participants (those who had solved the puzzle and those who did not) using three quantitative techniques of observational analysis (T-pattern detection, lag sequential analysis, polar coordinate analysis). Finally data was returned to a QUAL stage to interpret the results. The use of these three techniques allowed a detailed and in-depth analysis of the children's activity. Results reveal differences in the metacognitive abilities of the children that solved and didn't solve the puzzle. These results have important implications for educational practice.

## Introduction

Metacognitive skills (also called metacognitive regulation) refer to the processes that allow us to guide, regulate and supervise our own learning activities; that is, knowing how to learn and how and when to use a series of strategies to regulate our behavior. Generally metacognitive skills are divided into three component activities that are: (1) planning: it consists in the anticipation of the sequenced actions to be used to solve the task; (2) monitoring: it implies the review of the actions that are being carried out, their verification and rectification if necessary; (3) evaluation: it is about comparing the obtained result with the goals established at the beginning of the task. It also includes aspects related to the adequacy of the process followed (Flavell, 1992; Veenman et al., 2006; Chatzipanteli et al., 2014).

Metacognitive skills play an important role in a wide variety of activities including the exchange of verbal information, comprehension, reading, writing, attention, memory, problem solving, learning, or self-control. This helps to understand that metacognitive skills have been identified as a good predictor of academic success, even better than intelligence itself (Bryce et al., 2015; Nelson and Marulis, 2017; Mari and Saka, 2018). Thus, the level of metacognitive skills and the use that students make of them are differentiating variables between successful and unsuccessful students. Students who strategically use their metacognitive skills learn more and with less effort than those who do not use them; they detect and solve problems more easily and discover the best methods to reinforce what they have learned and transfer it to other contexts. This also makes them more involved and motivated toward learning, in addition to presenting greater self-efficacy (Chatzipanteli et al., 2014; Mari and Saka, 2018).

### Metacognitive Skills Development

Metacognitive skills emerge very early in life and develop during the following years (Chatzipanteli et al., 2014; Nelson and Marulis, 2017; Roebers, 2017). For example, it has been shown that children of 12 and 18 months, through their behaviors, show that they are already able to reflect on their own decisions to evaluate the accuracy of these and adapt their subsequent behaviors. Thus, they persist more in their behaviors after making a correct decision than when it is incorrect. Therefore, although complex forms of metacognition and verbal expression mature later in childhood, infants in their first year of life, through their behavior, show that they already estimate the accuracy of their simple decisions, monitor their errors, and use these metacognitive evaluations to regulate their subsequent behavior (Goupil and Kouider, 2016). Other studies have also shown that children of 18 months already use spontaneus strategies to correct their mistakes during problem solving (DeLoache et al., 1985). At 3 years, children are able to monitor their problem-solving behavior and at 4 years of using metacognitive processing in puzzle tasks (Sperling et al., 2000). Thus, there are various studies that show that, especially from 3 to 5 years of age, children show an important development in their metacognitive skills. Children are capable of solving their problems. They show different ways of planning, monitoring, and evaluation to do so, being able to monitor their behavior through different strategies (comments directed toward themselves, checking behaviors and error detection, behavior repetition to verify the accuracy of the result, use of gestures to support their activity) and establish behavior evaluation (including assessment of performance quality itself and evaluation when the task has been completed) (Whitebread et al., 2009; Bryce and Whitebread, 2012; Whitebread and Basilio, 2012; Whitebread and Pino-Pasternak, 2013). In short, the scientific evidence allows to affirm that the behavior of children already during the first year of life and during preschool years reveals basic forms of planning, monitoring and evaluation (Paulus et al., 2013; Chatzipanteli et al., 2014; Bernard et al., 2015; Roebers, 2017).

However, differences in the execution of metacognitive skills among children can be observed, which indicates the existence of different development rhythms of their metacognitive skills. Some children may not spontaneously acquire competent metacognitive skills. Veenman (2013) pointed out that those children who have metacognitive skills at their disposal but fail to produce them appropriately can be assisted by simple cues and reminders, provided by the context itself (for example, reminder posters) or by the teaching staff. However, children who do not have metacognitive skills may not benefit from simple cues and reminders, but can benefit from the effects of a specific further teaching and intervention, given that metacognitive skills are modifiable and teachable even in first ages (Whitebread and Basilio, 2012; Chatzipanteli et al., 2014).

### Metacognitive Skills Assessment

An important issue in the assesment of the development of early metacognitive skills as well as in their intervention proposals is to attend to the characteristics of the tasks that the child must solve, because at these early ages metacognitive skills are highly dependent on the context (Whitebread et al., 2009; Roebers, 2017). Thus, it is necessary that children are given the opportunity to launch their metacognitive skills by providing meaningful tasks for them, that is, that fit their interests and level of understanding (Chatzipanteli et al., 2014).

It was precisely methodological questions related to the tasks used during years in metacognitive skill research for children (besides theoretical issues) that made these early skills underestimated and even denied, affirming that metacognitive skills began to emerge at around 8–10 years. Recent research has allowed us to reject that position, allowing us to conclude that the characteristics of the tests and instruments used for their assessment underestimated these children's abilities by requiring a high verbal component, being that their linguistic development does not have to be at the same level as their metacognitive development (Whitebread et al., 2010; Mari and Saka, 2018). The indiscriminate use of self-report tools and laboratory studies with minors to assess metacognitive skills has also been criticized by experts (De la Fuente and Lozano, 2010; Mari and Saka, 2018). Current studies using an observational methodology, where children are studied in their own habitual context and their free, natural and spontaneous behavior is respected (without necessarily requiring explicit verbal response) have allowed to know that already at preschool ages, children use metacognitive skills to solve everyday problems (Whitebread et al., 2009; Escolano-Pérez et al., 2014; Nelson and Marulis, 2017; Mari and Saka, 2018).

Thus, natural contexts that have a meaning and purpose for children allow the implementation of metacognitive skills at a much earlier age than when they are exposed to artificial and meaningless environments. This makes systematic observation (characterized by allowing the capture of spontaneous behaviors as they occur in a natural context) the most appropriate, and often the only methodology that captures children competencies (Anguera, 2001; Bryce and Whitebread, 2012; Whitebread and Pino-Pasternak, 2013; Marulis et al., 2016; Escolano-Pérez et al., 2017).

Observational methodology nowadays is considered in itself as mixed-method because it integrates qualitative and quantitative elements in a QUAL-QUAN-QUAL macro stages (Anguera and Hernández-Mendo, 2016; Anguera et al., 2017, 2018a,b). In an initial qualitative stage an *ad hoc* observational instrument is constructed, totally adapted to the natural context and taking into account the objectives and observational design determined for its approach. The application of this observation instrument to the reality under study allows the registration of observational data to be obtained. Subsequently, the quantitative stage follows, in which the measurement parameters are obtained, the quality control of the observational data and its analysis is carried out. Finally, the interpretation of the results returns the process to the QUAL stage, permitting seamless integration.

One of the most exciting advances in observational methodology is the new possibilities of observational data analysis techniques. These techniques allow us a deeper study of spontaneous behaviors as they occur in a natural context. The most used techniques currently are: (1) *Temporal patterns (T-patterns) detection* (Magnusson et al., 2016; Casarrubea et al., 2018): a temporal pattern (T-pattern) is essentially a combination of events that occur in the same order with temporal distances between each other that remain relatively invariant in relation to the null hypothesis that says that each component is independent and is randomly distributed over time. The basic premise is that the interactive flow or chain of behavior is governed by structures of variable stability that can't be visualized by unaided observers but can be visualized by detecting these underlying T-patterns. This data analysis technique has been used successfully in studies developed in the field of social and human sciences, although in the specific area of education, despite its potential and richness of results, its use has been less common (Herrero-Nivela and Pleguezuelos Saavedra, 2008; Santoyo et al., 2017; Suárez et al., 2018). (2) *Lag sequential analysis* (Bakeman, 1978; Bakeman and Quera, 2011): this technical analysis calculates associated relationships between categories based on the calculation of observed and expected probabilities, and to compare them using a corrected binomial test, applying the correction of Allison and Liker (1982). Starting from a certain category (criterion or given behavior), it allows us to know what other categories (conditional behaviors) precede it in lag −1, −2, −3, etc. (retrospective lag sequential analysis) and/or what categories occur in lag +1, +2, +3, etc. (prospective lag sequential analysis) with an occurrence probability greater than being random. This technique has been used by different authors to detect and explore relationships between behaviors of different nature within the scope of social and human sciences. There are researchs that have analyzed the existing relationships between different behaviors carried out by students of different educational levels within the school context (Herrero-Nivela, 2000; Santoyo et al., 2017; García-Fariña et al., 2018), although this type of educational studies is less numerous than those performed in other human contexts; (3) *Polar coordinate analysis* (Sackett, 1980; Anguera, 1997): it is a data reduction technique that involves calculating the length and angle of vectors that reflect different relationships between a behavior of interest (known as a focal behavior) and other behaviors (known as conditional behaviors). Previously, calculating the adjusted residuals for the focal and conditional behaviors using lag sequential analysis is required. Relationship between the focal behavior and the corresponding conditional behaviors can be shown in a vector map with four quadrants. Each quadrant shows the type of relationship between these behaviors: Quadrant I (+ +): focal behavior activates and is activated by conditional behaviors; Quadrant II (– +): focal behavior inhibits but is activated by conditional behaviors; Quadrant III (– –): focal behavior inhibits and is inhibited by conditional behaviors; Quadrant IV (+ –): focal behavior activates but is inhibited by conditional behaviors. As with lag sequential analysis, despite the potential of polar coordinate analysis, it has been little used in the field of child development and learning (Herrero-Nivela, 2000; López Jiménez et al., 2016; Santoyo et al., 2017; Rodríguez-Medina et al., 2018).

So, observational methodology meets the rigorous standards of scientific inquiry while at the same time offers the flexibility needed in real-life settings. It is thus an ideal methodology for studying metacognitive skills of children while they are playing (Whitebread et al., 2009; Whitebread and Pino-Pasternak, 2013). Playing is an inseparable infantile life activity, reason why it is constituted as an indispensable mean for the observation of children's progress and development (Weisberg and Zosh, 2018).

Children games have an essential role in the evaluation of children since it constitutes a ubiquitous and universal aspect of childhood. Playing is the children's natural mode of expression and provides opportunities for their development and learning. This makes the game an essential tool for the children's teaching-learning process and for the systematic observation and analysis of their progress and development (Otsuka and Jay, 2017). Thus, the game can be considered a window through which we can systematically observe the affective, social, and cognitive functioning of children, thus allowing systematic observation of their metacognitive skills (Whitebread and Pino-Pasternak, 2013). The systematic observation of the child's behavior in this ludic context offers us great information and a variety of nuances that can allow us to describe, explain, and understand fundamental aspects of the child's development and learning that can not be appreciated with other methodologies. However, despite its importance for child development, there are few studies that carry out a validated and reliable assesment and intervention based on the game (Salcuni et al., 2017). Consequently, many authors claim the need for more observational studies to identify and analyze children's metacognitive behaviors in their own natural context of child development and learning, as well as through the performance of appropriate play tasks for their level of development (Whitebread et al., 2009; De la Fuente and Lozano, 2010; Whitebread and Basilio, 2012).

On the other hand, different authors (Whitebread and Coltman, 2011) state that the activities that favor the use of metacognitive skills in children, and therefore self-regulated learning, are those that allow and encourage the child to find his/her own way of solving the task; those in which each step to execute requires prior planning and later evaluation. In relation to all this, there are several authors who defend that puzzles, since they combine all the aspects that have just been mentioned (playful activity + the need to search in a specific way for resolution), constitute one of the best activities for solving children's problems promoting the implementation of metacognitive skills by children (Robson, 2015; Nelson and Marulis, 2017).

However, despite these methodological advances that have made possible to show that metacognitive skills appear from very early ages, there are still few empirical studies that evaluate metacognitive skills in child populations while being employed naturally, which would provide more adequate and valid measures of these infantile abilities (Paulus et al., 2013; Mari and Saka, 2018). Perhaps the scarcity of this type of research is due to the complexity and difficulties involved in working with very young participants, both due to their inherent characteristics (such as the high fluctuation of their motivation and their short periods of attention and activity on one task), as well as the ethical and legal issues involved in working with minors (for example, parents may be reluctant to allow children participation in research; De la Fuente and Lozano, 2010; Clark et al., 2013).

Thus, we find a small number of studies related to early metacognitive skills, but in addition, most of them focus on positive examples of metacognitive skills and frequently, only on one of them (Bryce and Whitebread, 2012), also reducing its analysis to simple frequencies of particular behaviors. All this prevents capturing all the information that involves the use of early metacognitive skills and offers a limited and misleading view of them.

### Aim

In order to overcome the limitations previously mentioned, the aim of this study is to analyze, complementing three observational data analysis techniques (T-pattern detection, lag sequential analysis and polar coordinate analysis), children records obtained to know the sequential and associative structure of the metacognitive skills that children of 5 years of age put into action during the resolution of a playful task (specifically, a puzzle). More precisely, it is interesting to know if there are different hidden structures in the use of metacognitive skills by children who solve the puzzle and those who do not, focusing on planning, monitoring, and evaluation metacognitive skills.

We hypothesize that children who solve the puzzle will use more advanced skills on planning that children who do not. That is, children who solve the puzzle will determine more accurately the steps to be taken to solve the puzzle. We predict that the determination of steps to solve the task will allow them to guide their actions. Consequently, these actions will be less hesitant and more fluid and autonomous. In addition, we expect them to be more aware and realistic than children who do not solve the puzzle when they evaluate their process followed to solve the task.

We postulate that the complementary use of these three data analysis techniques, a novel issue in the research of human behavior (Santoyo et al., 2017; Tarragó et al., 2017) and from our knowledge never used in the educational field in ages so early, will allow us to capture in depth and from different perspectives all the richness of children's metacognitive behavior.

We hope that results obtained will help education professionals to provide an educational response tailored to student needs, a key issue in any quality education system (Rodríguez-Dorta and Borges, 2017). This will help children to improve their metacognitive skills, which will imply enhancing their learning and academic success as well as their ability to solve problems throughout their lives and become competent citizens.

## Materials and Methods

### Design

We applied a mixed-method approach as ongoing method of assessment. The integration of qualitative and quantitative elements (characteristic of mixed-methods) was carried out from the “connect” option (Johnson et al., 2007). More exactly, we used observational methodology.

The observational design employed, according to the observational designs described by Anguera et al. (2018a), was Nomothetic/Punctual/Multidimensional (N/P/M), which was justified by the following arguments: *nomothetic* in regard to units of observation studied because we studied the metacognitive skills of 44 children playing individually; *punctual* (with intrasessional following) regarding the temporality of the assesment given that each participant was observed in a single session analyzing behavior succession, indicating their metacognitive skills within the session, and *multidimensional* in relation to the dimensionality of observed behavior because several metacognitive skills (planning, monitoring, and evaluation) were analyzed according to the theoretical model proposed by several authors (for example: Chatzipanteli et al., 2014) and the observation instrument reflected this multidimensional structure.

The observation of behavior in this study was scientifically rigorous because the behaviors observed as indicators of preschool metacognitive skills were fully perceivable and the observers had a non-participatory role. So, systematic observation was non-participative and active and behaviors observed were fully perceivable (Anguera, 2001; Bakeman and Quera, 2011; Shaughnessy et al., 2012).

### Participants

The sample used for this study was part of a wider research (Escolano-Pérez et al., 2017). The sample was composed by 44 Spanish children. They had a mean of 5.73 years old (SD Age = 0.30), 28 participants (63.6%) were women and 16 (36.4%) were men. They represented 95.65% of the last year of preschool students (last year of non-compulsory education in Spain) who attended the same educational city center in the north of Spain. All participants were of medium-high socioeconomic class, according to information offered by the center's management team.

The sample was a convenience sample formed by the preschoolers whose parents signed the informed consent authorizing the participation of their son/daughter in the study and who also fulfilled the following three inclusion criteria (Escolano-Pérez et al., 2017): (1) attendance at the targeted school since the first year of preschool education (age 3); (2) absence of the following disorders or risk factors: (a) gestational age <36 weeks and/or birth weight <2,000 g or significant pre-, peri-, or postnatal events; (b) medical/neurological conditions affecting growth, development, or cognition (e.g., seizure) and sensory deficits (e.g., vision or hearing loss); (c) neurodevelopmental disorders (e.g., attention-deficit hyperactivity disorder, autism spectrum disorder, language disorder); (d) genetic conditions or syndromes; (e) a first-degree relative with bipolar disorder, schizophrenia or related disorders; (3) an adequate IQ for their chronological age. The information to assess compliance with the first two criteria was provided by the children's parents. (In the informed consent that they had to sign authorizing the participation of their son/daughter in the study, questions were included in this regard). The information to assess compliance with the third inclusion criteria was obtained using the Spanish Battery of Differential and General Abilities Tests: level I (BADyG-I) (Yuste and Yuste, 2001).

### Instruments

Different instruments were used: those of the qualitative stage (Recording Instruments and Observation Instrument) and others specific to the quantitative phase (Data Analysis Instruments) that made up the assumed mixed methods perspective. Next, each of them is specified.

#### Recording Instruments

To record the playful activity of each of the participants, a Sony HDR-CX115 video camera was used.

To carry out the registration of the actions indicative of preschoolers' metacognitive skills, the free software Lince was used (Gabin et al., 2012). It was downloaded from http://lom.observesport.com/.

#### Observation Instrument

The observation of spontaneous behaviors in a natural context required the use of an observation instrument that was built *ad hoc*. We elaborated an observation instrument that combines a field format and systems of categories (Table 1). The choice of this type of instrument was justified by the multidimensionality of our observational design. The instrument was elaborated based on: (a) preliminary recordings of the reality object of study; (b) theoretical proposals about metacognitive skills (Chatzipanteli et al., 2014); (c) observation instruments used by other researchers to capture children's metacognitive skills (Muñoz, 2004; Whitebread et al., 2009); and (d) characteristics of the puzzle.

**Table 1:** Observation instrument.

**Criterion**	**Category systems**			**Category description**	**Example**	**Category code**
1. Planning	Inaccurate			The participant indicates where he/she is going to place the piece but he/she does it ambiguously and vaguely, without using specific spatial references (for example, without using references of the type “up/down,” “in the roof area,” “wall”).	The participant says: “*This* (points to piece 1) *goes here* (it indicates, without precision, a large space in the upper area of the table)”.	plim
	Accurate			The participant indicates in a concrete and precise way where he/she is going to place the piece and how, using concrete spatial references on what will be the location of the piece, its orientation and/or its relationship with the parts of the house.	The participant says: “*This* (points to piece 1) *I will rotate and put it up here* (points to a specific space in the upper area of the table), *with this peak up* (points to the right angle of the piece), *forming the roof of the house*”.	plp
	Does not know/Does not answer			The participant does not give any indication about how and where the piece will be placed.	The participant says: “*I do not know how the piece goes*”.	pln
2. Planned or moved piece	One			Piece of the puzzle marked with number 1	The participant says: “*This piece* the participant points to the piece marked with number (1) *I will put it down*”.	uno
	Two			Piece of the puzzle marked with number 2	The participant rotates the piece marked with the number 2.	dos
	Three			Piece of the puzzle marked with number 3	The participant tries to place the piece marked with number 3.	tres
3. Monitoring	Start	According to the planning	Success	The participant places a piece congruently to how he/she said he/she was going to do it before starting the task, being this location of the correct piece comparatively to the house model to be built.	The participant, during the planning, pointing out piece 1, its sides and vertices indicate that he/she will put the piece on the top of the table, with the vertex of the right angle upwards and the hypotenuse parallel to the bottom edge of the table. When he/she begins to perform the task, takes piece 1 and places it as he/she said. The result of this action is that piece 1 remains as a roof of the house.	acpla
			Error	The participant places a piece congruently to how he/she said he/she was going to do it before starting the task but the location of the piece is incorrect compared to the model of the house to be built.	The participant, during the planning, pointing piece 2 and its right angle, indicates that he/she will put the piece just at the bottom edge of the table, with its right angle downwards (the vertex of this angle touching the edge of the table) and its hypotenuse up (parallel to the edge of the table). When he/she begins to perform the task, the participant takes piece 2 and places it as he/she said. The result of this action is that piece 2 is not well located comparatively to the model of the house to be built. (It is displaced 180 degrees with respect to what it would be, for example, its correct location to form the roof).	acple
		Not according to planning	Success	The participant places a piece differently to how he/she said he/she was going to do it before starting the task but the location of the piece is correct comparatively to the house model to be built.	The participant, during the planning, pointing piece 2 and its right angle, indicates that he/she will put the piece just at the bottom edge of the table, with its right angle downwards (the vertex of this angle touching the edge of the table) and its hypotenuse up (parallel to the edge of the table). When the task begins, the participant takes piece 2 and places it in another way: he/she places the piece on the top of the table, with the vertex of the right angle upwards and the hypotenuse downwards, parallel to the lower edge of the table. The result of this action is that piece 1 remains as a roof of the house.	naca
			Error	The participant places a piece differently from how he/she said he/she was going to do it before starting the task, being also the location of the incorrect piece comparatively to the house model to be built.	The participant, during the planning, pointing piece 2 and its right angle, indicates that he/she will put the piece just at the bottom edge of the table, with its right angle downwards (the vertex of this angle touching the edge of the table) and its hypotenuse up (parallel to the edge of the table). When he/she begins to perform the task, the participant takes piece 2 and places it in another way: he/she places the piece in the upper area of the table, with its right angle facing left (looking toward the model house) and with its hypotenuse perpendicular to the edge of the table.	nace
	During	Trials	Success	The participant, after a first placement of the three pieces, takes one of them and changes its position or location without giving a reason for it, proving how and where to put it. The result of this new location of the piece is correct comparatively to the house model to be built.	Having placed the three pieces, so that piece 1 and piece 2 form a square as a facade of the house and piece 3 is located at the bottom of this square with the vertex of the right angle touching the square, the participant takes piece 3 and places it on top of the square, with its hypotenuse resting on the square.	taa
			Error	The participant, after a first placement of the three pieces, takes one of them and changes its position or location without giving a reason for it, proving how and where to put it. The result of this new location of the piece is incorrect compared to the house model to be built.	Having placed the three pieces, so that piece 1 and piece 2 form a square as a facade of the house and piece 3 is located at the bottom of this square touching with the vertex of the right angle the square, the participant takes piece 3 and places it on top of the square with the vertex of the right angle resting on the square.	tae
			Turns the figure correctly	The participant, after a first placement of the three pieces, rotates in any direction the figure that has formed with the three pieces (it implies therefore to rotate the three pieces together), so that by means of this action it obtains a correct figure comparatively to the model of house to build.	Once pieces 2 and 3 are placed forming a square as a facade and piece 3 is located in the lower part of this, the hypotenuse of the piece 3 touching the lower part of the square, the participant rotates the 3 pieces together 180° toward his/her left.	tga
			Turns the figure incorrectly	The participant, once the three pieces are placed, rotates in any direction the figure that has formed with the three pieces (it implies therefore to rotate the three pieces together), so that by means of this action it obtains an incorrect figure comparatively to the model of house to build.	Once pieces 2 and 3 are placed forming a square as a facade and the piece 3 located in the lower part of this, touching with the vertex of the right angle the lower part of the square, the participant rotates the 3 pieces together 90° toward his/her left.	tge
	End	Solves		The participant correctly solves the task, that is, realizes a house equal to the given house model placing the pieces correctly: He/she places a triangle with its right angle upwards and its hypotenuse downwards, forming the roof of the house. Under this triangle he/she places two triangles joined by their hypotenuses, forming a square as a facade. This square has one of its sides stuck to the hypotenuse of the upper triangle that it gave as a roof, leaving this triangle-roof supported and centered on the caudrado-facade of the house.	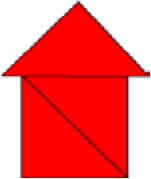	rs
		Does not solve		The participant does not solve the task or solves it incorrectly (that is, he does not manage to make a house equal to the given house model).	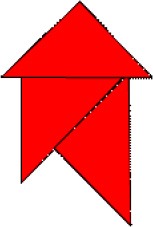	nrs
4. Evaluation	Correct justified			The participant issues a response about his/her successful resolution or not (good/bad) of the puzzle, which is consistent with the reality of the product obtained and issuing criteria based on those who make such a judgment. That is, the participant truthfully argues his/her answer.	Having correctly solved the puzzle, the participant says: “*I have done well because the two houses are the same: Look at the roof of this* (the participant points to the roof of the model house) *and this* (the participant points to the roof of the house built). *They are the same, they are up, and the walls of this one* (he/she points to the model house) *and the walls of my house … are the same, they are under the roof* ”.	coj
	Correct without justification			The participant issues a response about his/her successful resolution or not (good/bad) of the puzzle, being this congruent with the reality of the product obtained but without explaining or arguing the reasons that lead him/her to make such a decision.	Having correctly solved the puzzle, the participant says: “*It's okay*”.	cosj
	Incorrect			The participant issues a response about his/her successful resolution or not (good/bad) of the puzzle but this is inconsistent with the reality of the product obtained (regardless of whether he/she justifies his/her answer or not).	Having incorrectly solved the puzzle, the participant says: “*It's okay*”.	inc
5. Adult	Help			The adult participates in any phase of the task suggesting and/or offering the participant explicit clues to the completion of the task.	The adult says to the participant: “*Look closely at this piece, are you sure it is well placed*?”	aday
	Intervenes			The adult participates in any phase of the task encouraging, reinforcing the child to continue his/her task.	The adult says to the participant “*That's it, very well, continue*”.	adin

The observation instrument was made up of 5 dimensions or criteria. These criteria allowed to capture metacognitive skills that the child used during the process of solving the puzzle, meaning, planning (criterion 1), monitoring (criterion 3), and evaluation (criterion 4), in addition to the specific piece of puzzle he/she used each time in these skills (criterion 2). Capturing the participation of the adult accompanying the child was also possible with the last criterion (criterion 5). The criteria 1, 2, 3, and 4 had resulted in exhaustive and mutually exclusive category systems. The criterion 5 corresponded to a structure of field format as there was no closed set of coding possibilities.

#### Software Instruments

Four software programs were used: (a) SAS 9.1.3 (Schlotzhauer and Littell, 1997; SAS Institute Inc, 2004) to analyze observational data quality (intra and inter observational reliability); (b) THEME v.6 Edu (Magnusson et al., 2016) for the temporal patterns (T-patterns) analysis. This software was downloaded for free from http://patternvision.com; (c) GSEQ5, v.5.1 (Bakeman and Quera, 2011) for the lag sequential analysis. This software was downloaded for free from http://www.ub.es/comporta/sg/sg_s_download.htm; (d) HOISAN v.1.6.3.2 (Hernández-Mendo et al., 2012, 2014) for the polar coordinate analysis. It was downloaded for free from the online MenPas psychological evaluation platform (www.menpas.com).

### Procedure

The research project was approved by the school management team. Afterwards, teachers of the participants were informed about the aim and nature of the study. In adition, an informative meeting was held with the parents/guardians of the children. In this meeting they were given the informed consent that they had to return signed authorizing the participation of their son/daughter in the study and being recorded while playing. Parents/guardians were asked about the first two inclusion criteria in the sample: (1) child's attendance at the school since first year of preschool education (age 3) and (2) absence of certain disorders or risk factors in the child. Parents who did not attend the meeting were given information about the study and informed consent when they went to pick up their children at school.

Among those students whose parents signed the informed consent (a total of 44, representing 95.65% of all children who attended the last year of preschool), those who fulfilled the first two inclusion criteria were selected. All the children fulfilled them. To verify if these children also fulfilled the third inclusion criterion in the sample (an adequate IQ for their chronological age), the BADyG-I was administered collectively. This instrument-with adequate psychometric properties (Yuste and Yuste, 2001)-offered three scores: Verbal Intelligence, Non-Verbal Intelligence, and General Factor Intelligence. The 44 children evaluated obtained adequate scores for their chronological age, so all of them were part of the study sample.

**Figure 1:**
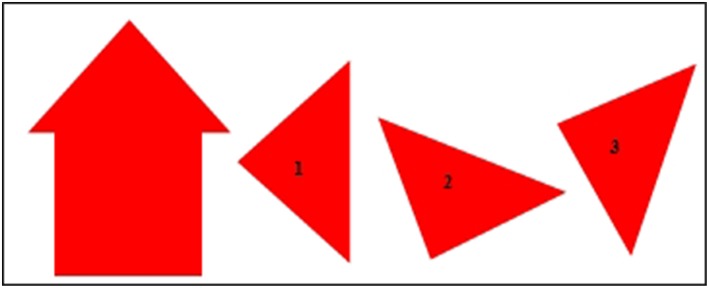
Puzzle: Model to be constructed by participants from the three pieces.

In accordance with the requirements of the observational methodology, exploratory, or preliminary observation sessions were held in order to gather information that would contribute to the construction process of the observation instrument as well as making subsequent decisions with guarantees. These preliminary sessions, as established by the observational methodology, were similar to the observation sessions themselves. They consisted of the following: an adult familiar with the child offered a puzzle to the participant in a classroom of the school (specifically in a classroom usually used by teachers of the center to perform individual or small group activities with children, being therefore a familiar classroom for participants). The concrete puzzle that was used in this study (and explained below) was elaborated based on the tangram and the puzzle used by Muñoz (2004) in his research with children of similar characteristics to those who composed the sample of this study. The puzzle consisted of the following (see Figure 1). The child had to build, from three equal pieces and in the form of a triangle (pieces 1, 2, and 3 of Figure 1) the house model that was presented to him/her (Figure 1). The three pieces were three right isosceles triangles. Their hypotenuse measured 19.8 cm and their legs 14 cm. The layout in which the participant was presented both the house model to be built and the 3 pieces with which to build it are shown in Figure 1. Both the house model and the pieces of the puzzle were made of Eva rubber foam, for being a flexible material and resistant to manipulation by children.

When the child was presented with the three pieces and the house model, he/she was told: “*You have to make a house equal to the model with these pieces* (the model and the pieces were pointed out)*, but before doing it you must think how you are going to do it. When you've thought about it, you'll tell me and then you'll do it*.” We made sure that he/she had understood what he/she had to do asking him/her: “*Have you understood?*”. In case the child answered yes, he/she was asked to explain it to verify that it was indeed like that. In case the child answered no, he/she was explained again a second time. (No participant needed to be repeated more explanations). Once it had been proven that the child understood what he/she should do, he/she was told: “*Very well, remember: first think how to do it, second tell me and third, do it*.” The time available for the child to do any of these three actions (think about how he/she was going to solve the puzzle, tell it, and execute it) was not limited.

The moment in which the child began to tell how he/she was going to solve the puzzle was considered the beginning of the observation session. (This explanation given by the child on how he/she was going to solve the puzzle allowed to evaluate the metacognitive planning skill). Once the child had finished this explanation, he/she was told: “*Now you can do the puzzle, telling me in a loud voice what you do and why you do it*.” (With this we evaluated not only the monitoring strategy but also whether it fitted the plan and if the child was able to realize his/her mistakes in the planning and the way to solve them). When the child finished the task he/she was asked if this was good and why so that he/she could evaluate if his/her evaluation strategy was consistent not only with the final result but also with the whole process carried out. When the child finalized his/her answers to these questions, it was considered the end of the observational session. All the observational sessions were recorded so that they could be analyzed later.

There were five preliminary sessions in which five children, individually, played with the puzzle. These preliminary sessions, which lasted an average of 9.45 min, were video recorded for later viewing. With the information gathered in these preliminary sessions about the metacognitive skills used by children to solve the puzzle, in addition to the information extracted from theoretical models about metacognitive skills and from observation instruments constructed by other authors for similar purposes, the observation instrument was constructed. It was necessary to build and adjust different intermediate versions until reaching the final version (Table 1).

The observation sessions themselves had an average duration of 9.31 min. All the sessions were video recorded.

The video recordings were imported into the Lince software and coded using the *ad hoc* observation instrument. Two observers (who were expert in observational methodology, child development and learning, and metacognitive skills) coded. The data entered included information on the frecuency and order of behaviors. In accordance with the type of data proposed by Bakeman (1978), the data were concurrent and event-based (Type II). This type of data was consistent with the multidimensional nature of the design.

Observational data quality was assessed qualitatively through consensual agreement and quantitatively by calculating intra- and inter-observer reliability. In the first four sessions to be codified, the consensual concordance between the two observers was applied. Then, observer 1 coded all the remaining sessions and calculated intra-observer reliability in 11 sessions. Observer 2 codified another 11 sessions that were used to calculate interobserver reliability with the corresponding records made by observer 1. Intra and interobserver reliability were calculated through intraclass correlation coefficient using SAS 9.1.3 software. In all cases good reliability was obtained (intraclass correlation coefficient ≥.90).

### Data Analysis

We used three techniques of data analysis in order to answer to the aim of this study: (1) T-patterns detection; (2) Lag sequential analysis; and (3) Polar coordinate analysis.

#### T-patterns Detection

In agreement with the aim of the study, the detection of T-pattern focuses on the analysis of the whole recording of the puzzle. Therefore, the files with the encoded data of each participant were concatenated into a single multi-sample file.

The following search parameters were set in THEME v.6 Edu for the detection of T-patterns (for further information see Reference Manual, PatternVision Ltd and Noldus Information Technology bv, 2004): (a) frequency of occurrence ≥7; (b) level of significance *p* < 0.005; (c) deactivation of fast requirement at all levels and selection of free heuristic critical interval setting; (d) validation of results through randomization of data on five occasions (i.e, detected T-patterns were only accepted if THEME detects them among all the additional randomly generated relationships).

Once the T-patterns were obtained, statistical analysis was carried out on them according to the aim of the study. It was determined to look for patterns that were significantly more frequent in children who did solve the puzzle and those who were significantly more frequent in children who did not solve the puzzle. Once these patterns were obtained, through the application of qualitative filters, those of greatest interest for the study objective were selected; that is, the patterns that reflected the complete or almost complete resolution process, implying: (a) that they started with some category related to *Planning* [“Inaccurate planning” (*plim*), “Accurate planning” (*plp*), and “Does not know/Does not answer” (*pln*), regardless of which *piece was planned* (*uno, dos* or *tres*)] and (b) finished by some category related to *End* [i.e., categories “Solves” (*rs*) and “Does not solve” (*nrs*)] or by some category related to *Evaluation* [“Correct evaluation without justifying” (*cosj*), “Correct evaluation justified” (*coj*), and “Incorrect evaluation” (*inc*)].

#### Lag Sequential Analysis

In this study, according to the aim, a lag sequential analysis was calculated for each children group (children who solved the puzzle and those who did not). Behaviors selected as criterion behaviors in the group of children who solved the puzzle were: (a) all the categories related to the metacognitive ability of *Planning*, that is: “Inaccurate planning” (*plim*), “Accurate planning” (*plp*), and “Does not know/Does not answer” (*pln*), regardless of which piece was planned (*uno, dos* or *tres*); (b) the *Monitoring* category “Solves” (*rs*) and (c) all the categories referred to *Evaluation*: “Correct evaluation without justification” (*cosj*), “Correct evaluation justified” (*coj*) and “Incorrect evaluation” (*inc*). In the group of children who did not solve the puzzle, these same categories were used as criterion behaviors, except “Solves” (*rs*) that was replaced by “Does not solve” (*nrs*). In both groups of children, as conditional behaviors, we selected all the categories of the observation instrument. Given the psychological meaning of each category selected as criterion behavior (and consequently the moment of childhood activity in which each of them could appear generating prospective or retrospective patterns), from multievent sequential data (using lag sequential analysis lexicon), we looked at: 10 prospective lags (from +1 to +10 lags) that occurred immediately after the criterion behavior referred to *Planning* [“Inaccurate planning” (*plim*), “Accurate planning” (*plp*) and “Does not know/Does not answer” (*pln*)]; 10 retrospective lags (from −10 to −1 lags) that occurred immediately before the criterion behavior “Solves” (*rs*) or “Does not solve” (*nrs*) and 10 prospective lags (from +1 to +10 lags) that occurs immediately after these criterion behavior; and 10 retrospective lags (from −10 to −1 lags) that occurred immediately before the criterion behavior referred to *Evaluation* [“Correct evaluation without justification” (*cosj*), “Correct evaluation justified” (*coj*), and “Incorrect evaluation” (*inc*)]. The level of significance was set at *p* < 0.05. We used software program GSEQ5, v.5.1.

#### Polar Coordinate Analysis

Given the aim of this study, the analysis of polar coordinates was made, on the one hand, with the observational data of children who solved the puzzle and, on the other, with the data of children who did not solve the puzzle. We used the software program HOISAN v.1.6.3.2. The categories chosen as focal behaviors were: all the categories related to the metacognitive ability of *Planning* [“Inaccurate planning” (*plim*), “Accurate planning” (*plp*), and “Does not know/Does not answer” (*pln*)]; the category of “Solves” (*rs*) or “Does not solve” (*nrs*) depending on whether it was a group of children or another; and the *Evaluation* categories [“Correct evaluation without justification” (*cosj*) and “Incorrect evaluation” (*inc*)]. The categories chosen as conditional behaviors were all the categories that made up the observation instrument.

Given the aim of our study, which sought to analyze the sequential and associative structure of the metacognitive skills that children put into action during the resolution of the puzzle, the results obtained in quadrant I and IV were considered, quadrants in which the focal behavior activates conditional behaviors. [This is why the category “Correct evaluation justified” (*coj*) was not considered as a focal given that it implied the end of the game and therefore, it can not activate any category].

## Results

### T-patterns Detection

Table 2 shows 11 T-patterns obtained in the group of children that solved the puzzle, as well as the frequency of each of them and their length. These patterns appear ordered from greater to shorter length.

**Table 2:** T-patterns obtained in the group that solved the task.

T-patterns	*N*	Length
((( plim,tres acpla,uno )( taa,dos rs ))( adin cosj ))	7	6
( plp,uno ((( plim,tres acpla,uno )( taa,dos rs )) adin ))	7	6
( plim,dos (( plim,tres acpla,uno )( taa,dos rs )))	7	5
(( plp,uno plim,tres )(( acpla,uno taa,tres ) rs ))	7	5
( plim,tres ( acpla,uno ( taa,tres rs )))	8	4
(( plp,uno ( acpla,uno rs )) coj )	7	4
(( plim,tres acpla,uno )( rs coj ))	7	4
( plim,dos (( acpla,uno taa,tres ) rs ))	9	4
((( plim,dos acpla,uno ) taa,dos ) rs )	7	4
( plp,uno ( acpla,dos rs ))	7	3
( plp,uno ( acpla,tres rs ))	7	3

Patterns obtained show that children can reach the correct resolution of the puzzle starting from a precise (*plp*) and imprecise (*plim*) planning. Specify that precise planning (*plp*) always refers to piece one (*plp,uno*), while imprecise planning (*plim*) refers either to piece two (*plim,dos*) or piece three (*plim,tres*). The number of patterns obtained with precise planning (*plp*) (5 patterns) is very similar to that obtained with imprecise planning (*plim*) (6 patterns). Within these last patterns generated by imprecise planning (*plim*), the same number of patterns are obtained with piece two (*plim,dos*) (3 patterns) as with piece three (*plim,tres*) (3 patterns).

Although these patterns are typical of children who solved the task, only in three patterns does the evaluation (*cosj* and *coj*) appear. Therefore, the evaluation metacognitive skill is the one least used by participants. However, these evaluations are always correct (*cosj* and *coj*), and there are more numerous patterns that contain justified evaluations (*coj*, 2 patterns) than those that contain unjustified evaluations (*cosj*, 1 pattern).

The patterns obtained in the group of children that solved the task show low adult participation (*adin*). Adult intervention (*adin*) only appears as part of two patterns and also does it once the child has already solved the task (*rs*). Therefore, it seems that the adult would intervene with the intention of encouraging the evaluation of the child, although it does not always seem to achieve it: in a pattern, after the adult intervention (*adin*) there is a correct, although unjustified, evaluation of the child (*cosj*) but in another pattern, this does not happen (after *adin* no category happens).

Table 3 shows the only pattern obtained in the group of participants who did not solve the puzzle, its frequency and length.

**Table 3:** T-pattern obtained from the group that did not solve the task.

**T-pattern**	**N**	**Length**
((( plim,dos nrs )( tae,tres aday ))(( tae,dos aday ) nrs ))	7	7

Participants who did not solve the task planned in an imprecise way the location of piece two (*plim,dos*) and during its execution they carried out unsuccessful trials (*tae*). These children were not able to successfully perform the task despite receiving help from the adult (*aday*) during several moments of their execution. Thus, neither their planning metacognitive skill nor their monitoring metacognitive skill show adequate indicators to solve the task. There are no indicators of the evaluation metacognitive skill.

### Lag Sequential Analysis

Tables 4, 5 collect the retrospective and prospective patterns obtained in the group of participants that solved the puzzle. Patterns of interest are highlighted according to the aim of the study, which as previously indicated are the patterns that reflect the complete or almost complete resolution process. Therefore, according to the retrospective patterns, interest patterns are those that begin on some category related to *Evaluation* [“Correct evaluation without justification” (*cosj*), “Correct evaluation justified” (*coj*), and “Incorrect evaluation” (*inc*)] or by the *End* execution of the task [“Solves” (*rs*)] and finalize by some category related to *Planning* [“Inaccurate planning” (*plim*), “Accurate planning” (*plp*), and “Does not know/Does not answer” (*pln*)]. Considering the prospective patterns, those patterns that begin with one of the categories referred to *Planning* and *End* either by “Solves” (*rs*) or by some category related to *Evaluation* are of interest.

**Table 4:** Retrospective patterns obtained in the group of participants who solved the task.

Lag −10	Lag −9	Lag −8	Lag −7	Lag −6	Lag −5	Lag −4	Lag −3	Lag −2	Lag −1	Criterion behavior
			adim (2.27)			tae,dos (2.07)	acpla,dos (3.84) tae,tres (3.27)	acpla,tres (2.67)taa,dos (3.35)taa,tres (4.76)	acpla,tres (3.05) acpla,uno (3.74) taa,dos (6.24) taa,tres (5.36)	rs
		acpla,uno (2.13)**plp,uno (2.64)**	**plim,dos (2.74)**	**acpla,tres (1.99)plim,tres (2.74)**	**acpla,tres (2.0) acpla,uno (2.81) acple,uno (2.0) taa,dos (2.05)**	**acpla,dos (2.1)taa,uno (2.1)**	**acpla,tres (4.41) acpla,uno (2.15)**	**rs (5.36)**	**adin (2.55)** **inc (3.12)** rs (2.06)	**coj**
				coj (2.3)	taa,tres (2.23) tae,tres (2.08)	taa,tres (3.01)	nrs (2.12) taa,dos (2.13) taa,tres (2.48) tge (2.12)	inc (2.41)rs (4.29)	adin (2.62) rs (4.11) tga (2.66)	cosj

*Bold values indicate patterns of interest according to the aim of the study*.

**Table 5:** Prospective patterns obtained in the group of participants who solved the task.

**Criterion behavior**	**Lag +1**	**Lag +2**	**Lag +3**	**Lag +4**	**Lag +5**	**Lag +6**	**Lag +7**	**Lag +8**	**Lag +9**	**Lag +10**
rs		coj (6.61)cosj (6.11)								
**plp,uno**	**plp,dos (3.95) plim,dos (3.08)**	**plim,tres (6.01)plim,dos (3.08)nace,tres (2.17)**	**acpla,uno (3.07)**	**acpla,uno (3.82)nace,uno (1.98)**	**acpla,dos (1.98) naca,tres (1.98) nace,dos (1.98) nace,tres (1.98)**	**acpla,tres (2.82)acple,dos (2.82)tae,uno (1.98)**	**nrs (2.79)**	**coj (2.42)**	**taa,tres (3.28) inc (2.28)**	**rs (2.14)** taa,tres (2.14)
**plp,dos**	**plp,tres (6.51) nace,tres (3.72)**	**plp,uno (6.27)plim,uno (3.58)**	**nace,uno (3.21) aday (2.17)** **adin (2.17)**	**acpla,dos (3.66)naca,tres (3.21)nace,dos (2.06)**	**acpla,tres (3.29) tae,uno (3.21)**	**acpla,uno (4.56)acple,uno (3.21)taa,dos (2.17)**	**rs (2.42)**			aday (2.32) adin (2.32)
**plp,tres**	**plp,uno (10.15)**	**plp,dos (4.82)**	**nace,tres (2.91) acpla,dos (2.47)**	**acpla,tres (2.28)**	**acple,uno (4.93) nace,uno (4.33)**	**rs (3.26)**	**adin (2.03)**	**cosj (2.5)**	aday (2.6)	
plim,uno	plim,dos (4.85)	aday (3.16)plim,tres (2.35)	plim,dos (3.21)	plim,tres (3.21)acple,uno (2.06)	acple,tres (2.17) acple,uno (2.06) taa,uno (3.21)	acpla,dos (3.21)acple,tres (2.06)aday (2.06)nrs (2.17)	acpla,uno (3.11)			tae,tres (3.03)
plim,dos	plim,tres (6.57)	adin (3.29)acpla,uno (2.45)	taa,dos (1.98)	acple,tres (3.1)	tae,tres (3.97)	tae,dos (2.21)				
**plim,tres**	**acpla,uno (4.63)** **acple,dos (2.05)** **plim,uno (2.05)**	**acpla,uno (3.82)acple,dos (2.21)**	**acple,tres (3.37)**	**tae,tres (2.4)**	**taa,dos (3.5) tae,dos (2.52)**		**taa,tres (3.2)**	**rs (2.47)**		

*Bold values indicate patterns of interest according to the aim of the study*.

Tables 6, 7 show the retrospective and prospective patterns obtained in the group of participants who did not solve the puzzle. As in the previous case, patterns of interest are highlighted according to the aim of the study. The criteria that justify their consideration as patterns of interest are the same as those just explained for the group of participants that solved the task, except that when dealing with the patterns of children who did not solve the task, the “Solves” category (*rs*) is replaced by “Does not solve” (*nrs*).

**Table 6:** Retrospective patterns obtained in the group of participants who did not solve the task.

**Lag −10**	**Lag −9**	**Lag −8**	**Lag −7**	**Lag −6**	**Lag −5**	**Lag −4**	**Lag −3**	**Lag −2**	**Lag −1**	**Criterion behavior**
tae,tres (1.98)	adin (2.91)		tae,dos (2.38) **plim,tres (2.33)**	**plim,dos (3.04)**	**tae,dos (2.0)**		**acpla,uno (2.68) tae,dos (2.28)**	**tae,tres (4.3)tae,dos (3.81)** **acple,dos (3.02)taa,dos (2.32)**	**tae,tres (4.61) aday (2.95) tae,dos (2.71) acple,tres (2.34)**	**nrs**
tga (4.31)	acpla,dos (4.31) acpla,uno (2.89) cosj (2.89) taa,tres (2.88)	acple,uno (4.31)acple,dos (2.89)	acple,tres (4.31) **plp,dos (4.31)** cosj (2.89) taa,uno (2.89)	**plp,tres (4.31)**	**naca,uno (4.33) plp,uno (2.9)** **taa,tres (2.24)**	**nace,dos (4.33)tae,uno (2.9)**	**nace,tres (4.33) acpla,tres (2.24)**	acpla,uno (4.34)**nrs (3.86)**		**coj**
taa,dos (2.21)	tae,tres (3.04) **plim,dos ( 2.33)**		**acpla,dos (2.33) taa,dos (2.21)**			**taa,uno (3.58)taa,dos (2.86)tae,tres (2.0)**	**tga (5.08)** **tae,tres (2.47)** **taa,uno (2.34)**	**nrs (3.94)taa,tres (2.35)**	**nrs (2.5)** **adin (2.06)**	**cosj**
**plim,uno (2.57)plp,tres (2.42)**	**plim,tres (4.89) inc (2.5)**	**plim,dos (3.43)acpla,tres (2.42)**	**plim,dos (2.5) coj (2.42)**	**acple,tres (2.57)acple,uno (2.42)coj (2.42)**	**acpla,uno (3.45) acple,dos (3.45)**	**acple,uno (3.45)acle,dos (2.04)**	**acple,tres (2.04)**	**inc (3.04)nrs (2.03)**	**nrs (4.54)**	**inc**

*Bold values indicate patterns of interest according to the aim of the study*.

**Table 7:** Prospective patterns obtained in the group of participants who did not solve the task.

**Criterion behavior**	**Lag +1**	**Lag +2**	**Lag +3**	**Lag +4**	**Lag +5**	**Lag +6**	**Lag +7**	**Lag +8**	**Lag +9**	**Lag +10**
nrs	inc (3.93) cosj (2.36)	coj (2.1) cosj (3.05)								
**plp,uno**	**plp,dos (3.8) acpla,uno (3.09) acpla,tres (2.51)**	**plim,tres (3.07)** **acpla,dos (2.5)**	**acpla,uno (3.9) acple,uno (3.77)**	**nrs (3.62)**	acple, dos (3.96) **coj (3.77)** acple,tres (2.16)	aday (2.31)	plp,dos (4.0)		acpla,tres (3.98)	
**plp,dos**	**plp,tres (6.66)**	**plp,uno (4.53)** **acpla,uno (3.33)**	**acpla,tres (2.65) acple,dos (2.04)**	**acpla dos (3.97)** **acpla,tres (3.97)**	**acple,uno (3.97)**	**nrs (2.51)** taa,dos (2.29)	**coj (3.14)** taa,dos (2.64)		plp,dos (5.72) taa,dos (2.63)	adin (2.07) plim,dos (3.92)
**plp,tres**	**plp,uno (5.04) acpla,uno (2.0)**	**naca,uno (3.52)** **acpla,tres (2.77)**	**acpla tres (5.0) acpla dos (3.51) nace dos (3.51)**	**naca,tres (5.14)** **acple,uno (3.51)**	**nrs (2.85)**	taa,dos (4.38) **coj (3.49)**	tae,tres (2.09)	plp,dos (5.12) tae,dos (2.21)	plim,dos (3.48) adin (2.82) taa,dos (2.28)	coj (3.46) acpla,uno (2.27)
plim,uno	plim,tres (5.82) acpla,tres (2.92) plim,dos (2.07)	plim,dos (6.42) plp,tres (2.9)	acpla dos (2.89)	acpla,uno (4.27) naca,uno (2.89) acple,tres (2.4)	nace,dos (4.31) acple,tres (4.27) acpla,tres (2.23)	acple,uno (4.29) naca,tres (4.29) acple,dos (2.61)	taa,tres (4.29)	inc (2.39)	aday (2.19)	inc (2.58)
**plim,dos**	**plim,tres (3.07) plim,uno (2.87) plp,uno (2.55) acpla,uno (2.13)**	**acple,dos (3.13)** **acpla,uno (3.06)**	**acple,tres (3.49) acple dos (3.27) nace,tres (2.96)**	**acple,dos (3.21)**	**naca,tres (2.96) taa,dos (2.23)**	**nrs (2.2)**	**inc (2.34)**	taa,dos (2.21)		taa,tres (4.15)
plim,tres	plim,dos (5.63) acple,dos (4.62) acpla,dos (3.25)	nace,tres (3.4) acpla,uno (2.66) acple,tres (2.2)	acpla,uno (3.36) acple,tres (2.87) acple,dos (2.32)	taa, uno (3.38) acple, tres (2.87) acple, dos (2.64) acpla, tres (2.19)	acple,uno (2.19) acple,dos (2.04)			aday (2.51)	inc (3.81) tae,tres (3.25)	

*Bold values indicate patterns of interest according to the aim of the study*.

In the group of participants who solved the task, taking into account the patterns obtained in the retrospective perspective (Table 4), it can be seen that: (a) taking as criterion behavior “Correct evaluation justified” (*coj*), 64 patterns are obtained up to the delay−8 which contains an “Accurate planning of piece number one” (*plp,uno*).

Considering the prospective patterns of this same group of participants (Table 5), it is appreciated that all the precise planning (*plp*), independently of the piece to which they refer (*uno, dos*, or *tres*) generate patterns of interest. Taking into account the different bifurcations that are generated in some of the delays, a total of 508 possible patterns of this type are obtained: 288 generated from “precise planning of piece number one” (*plp,uno*); 216 generated from “precise planning of piece two” (*plp,dos*) and 4 generated from “precise planning of piece three” (*plp,tres*). Only these four patterns generated by “precise planning of piece three” (*plp,tres*) contain aspects related to evaluation, being correct but not justified (*cosj*).

Although sometimes the child starts planning inaccurately (*plim*), he/she can solve the task (*rs*), but not evaluate it. This happens only when this imprecise planning (*plim*) affects piece three (*plim,tres*) (not piece one or two, which do not generate patterns of interest). In particular, imprecise planning of piece three (*plim,tres*) generates 12 patterns.

Regarding the patterns obtained in the group of children who did not solve the task, taking into account the retrospective perspective (Table 6), it can be seen that all categories considered as criteria [“Does not solve” (*nrs*) and all those referred to *Evaluation* (*coj, cosj, inc*)] generate patterns of interest (a total of 510 patterns). As a whole, these patterns show that although participants did not solve the task, previously used planning, both precise (*plp*) and imprecise (*plim*), and both types of planning affecting any of the three pieces involved (*uno, dos, tres*).

The patterns generated by the category “Correct evaluation justified” (*coj*, 12 patterns) indicate that children were able to correctly evaluate and justify their result, even though previously they could not solve the puzzle successfully. And this happened even though they previously made precise planning (*plp*) of the three pieces that make up the puzzle.

However, the patterns generated by the category “Does not solve” (*nrs*), compared to the previous ones [those generated by “Correct evaluation justified” (*coj*)], show us important differences in the metacognitive skills of children: participants who did not solve the task and did not evaluate it, previously made plans that were imprecise (*plim*). On the other hand, just as it has been explained in the previous paragraph, the children who did not solve the task but correctly evaluated and justified their result, had made previous precise plans (*plp*).

It is noteworthy that, in relation to the evaluation categories, as the quality of children's evaluation increases (“incorrect” -*inc*-, “correct without justifying” -*cosj*-, and “correct justified” -*coj*-) less number of patterns are generated (*inc*, 394 patterns; *cosj*, 72 patterns; *coj*, 12 patterns).

In regard to the prospective patterns (Table 7), certain similarities are detected with the group of participants that solved the task in that all precise planning (*plp*) generated interest patterns; although a smaller number of patterns is generated. In this case, a total of 44 patterns are generated, distributed as follows: 12 from “precise planning of piece one” (*plp*,*uno*); 8 from “precise planning of piece two” (*plp,dos*); and 24 from “precise planning of piece three” (*plp,tres*).

In addition, corroborating the information previously exposed referred to the retrospective patterns generated by “correct evaluation justified” (*coj*), the following is appreciated. Precise planning (*plp*) with any of the 3 puzzle pieces gives rise to patterns that show that although children did not solve the task correctly, they were able to issue a justified correct evaluation (*coj*).

In the patterns generated from imprecise planning (*plim*), differences appear between groups of children. In the group of participants that did not solve the task only the “imprecise planning of piece two” (*plim,dos*) generates patterns (48 patterns). Remember that imprecise planning only generates patterns with piece three (*plim,tres*) in the other group of participants (participants that solved the task).

### Polar Coordinate Analysis

**Figure 2:**
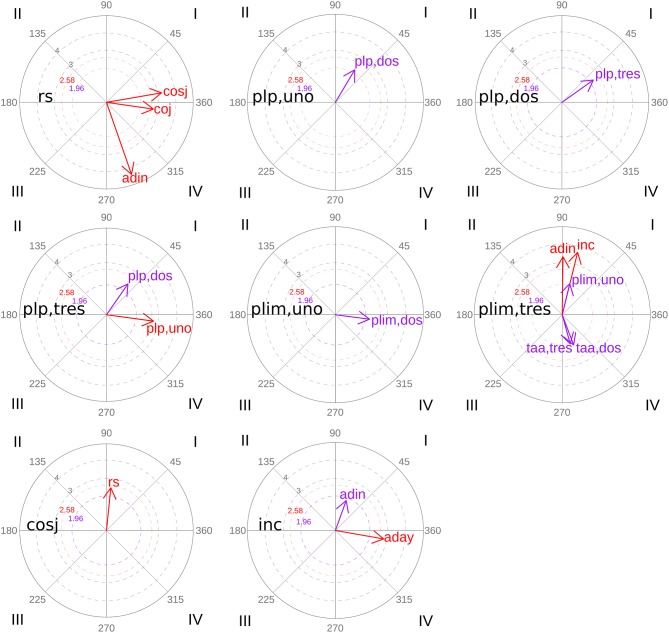
Polar coordinate vector maps: group of participants that solved the task.

Table 8 and Figure 2 show the significant results of polar coordinate analysis and its vector maps for the group of participants that solved the task.

**Table 8:** Polar coordinate analysis results: group of participants that solved the task.

Focal behavior	Categories	Quadrant	Prospective perspective	Retrospective perspective	Radius	Angle
rs	cosj	I	3.17	0.55	3.22 (^*^)	9.8
	coj	IV	2.7	−0.39	2.73 (^*^)	351.81
	adin	IV	1.45	−4.16	4.4 (^*^)	289.16
plp,uno	plp,dos	I	1.1	1.85	2.15 (^*^)	59.25
plp,dos	plp,tres	I	2.06	1.46	2.53 (^*^)	35.42
plp,tres	plp,dos	I	1.41	2.03	2.47 (^*^)	55.15
	plp,uno	IV	3.09	−0.42	3.12 (^*^)	352.26
plim,uno	plim,dos	IV	2.21	−0.29	2.23 (^*^)	352.59
plim,tres	plim,uno	I	0.48	2.01	2.07 (^*^)	76.47
	inc	I	1	4.04	4.16 (^*^)	76.15
	adin	I	0.05	3.75	3.75 (^*^)	89.23
	taa,dos	IV	0.74	−1.95	2.09 (^*^)	290.66
	taa,tres	IV	0.52	−1.89	1.96 (^*^)	285.34
cosj	rs	I	0.28	2.80	2.82 (^*^)	84.20
inc	adin	I	0.7	1.94	2.06 (^*^)	70.2
	aday	IV	3.13	−0.55	3.17 (^*^)	350.08

* *Significant relationships (p < 0.05) between the focal behavior and conditional behaviors*.

Among the results obtained, the following aspects stand out (Figure 2). Task resolution (*rs*) activates correct evaluations, regardless of whether they are justified (*coj*) or not (*cosj*). Precise planning (*plp*) is activated mutually, except for precise planning with piece three (*plp,tres*) although it activates precise planning of piece one (*plp,uno*), this inhibits the previous one. Incorrect evaluation (*inc*) activates adult participation (*adin* and *aday*), although depending on what type of adult participation is concerned, this can activate the incorrect evaluation (case of *adin*-adult intervention-) or inhibit it (case of *aday*-adult help-).

**Figure 3:**
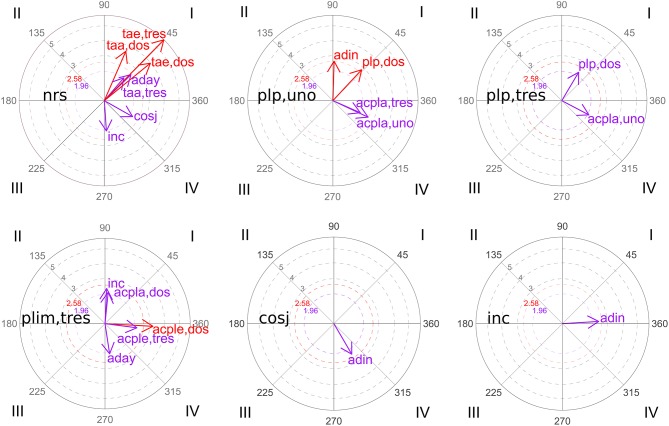
Polar coordinate vector maps: group of participants that did not solve the task.

Table 9 and Figure 3 represent the significant results of polar coordinate analysis and its vector maps for the group of participants who did not solve the task. The non-resolution of the task (*nrs*) activates trials with piece two and piece three, these trials being able to imply both correct (*taa,dos* and *taa,tres*) and error (*tae,dos* and *tae,tres*) outcomes. All these actions in turn also activate the non-resolution of the task (nrs). The same happens with adult help (*aday*): the non-resolution of the task (*nrs*) and adult help (*aday*) activate each other. On the other hand, although the non-resolution of the task (*nrs*) activates evaluation that is both correct without justifying (*cosj*) and incorrect (*inc*), both evaluations inhibit that category. When referrals to evaluation are considered as focal behaviors (“correct evaluation without justifying” (*cosj*) and “incorrect evaluation” (*inc*)] these activate adult intervention (*adin*).

**Table 9:** Polar coordinate analysis results: group of participants that did not solve the task.

Focal behavior	Categories	Quadrant	Prospective perspective	Retrospective perspective	Radius	Angle
nrs	taa,dos	I	1.44	3.3	3.6 (^*^)	66.43
	taa,tres	I	1.31	1.53	2.01 (^*^)	49.4
	tae,dos	I	3.08	2.5	3.97 (^*^)	39.08
	tae,tres	I	3.98	4.06	5.69 (^*^)	45.61
	aday	I	1.78	1.74	2.49 (^*^)	44.28
	cosj	IV	1.89	−1.07	2.17 (^*^)	330.52
	inc	IV	0.14	−2.03	2.03 (^*^)	273.93
plp,uno	plp,dos	I	1.9	2.04	2.78 (^*^)	47.03
	adin	I	0.08	2.6	2.6 (^*^)	88.26
	acpla,uno	IV	2.31	−1.08	2.56 (^*^)	334.89
	acpla,tres	IV	1.8	−0.79	1.97 (^*^)	336.2
plp,tres	plp,dos	I	1.13	1.9	2.21 (^*^)	59.25
	acpla,uno	IV	1.82	−0.95	2.05 (^*^)	332.33
plim,tres	acpla,dos	I	0.21	2.1	2.11 (^*^)	84.25
	inc	I	0.11	2.35	2.35 (^*^)	87.3
	acple,dos	IV	3.21	−0.18	3.21 (^*^)	356.79
	acple,tres	IV	2.13	−0.28	2.15 (^*^)	352.57
	aday	IV	0.31	−2.01	2.04 (^*^)	278.66
cosj	adin	IV	1.18	−2.01	2.33 (^*^)	300.35
inc	adin	I	2.38	0.15	2.39 (^*^)	3.49

* *Significant relationships (p < 0.05) between the focal behavior and conditional behaviors*.

Among all these results that allow us to respond to our objective, we first compare the temporal patterns (T-patterns) and the sequential patterns and then, we compare both with the significant results of the polar coordinate analysis (associative structures) in order to know if they can be complementary.

Concerning the T-patterns and the sequential patterns, we can distinguish between those structures that can be considered complete (they contain information referring to the three metacognitive skills analyzed: planning, monitoring, and evaluation) and those that can be considered incomplete (only referred to the metacognitive skills of planning and monitoring).

The number of structures of each obtained typology with lag sequential analysis and with T-pattern detection is shown in Table 10.

**Table 10:** Classification of the sequential and associative structures obtained with lag sequential analysis and T-pattern detection.

Group of participants	Data analysis technique	Pattern start	N° complete prospective patterns	N° incomplete prospective patterns	N° complete retrospective patterns	N° incomplete retrospective patterns
Solves	T-pattern detection	plp,uno	1	4	–	–
		plim,dos	0	3	–	–
		plim,tres	2	1	–	–
	Sequential analysis	coj	–	–	64	0
		plp,uno	0	288	–	–
		plp,dos	0	216	–	–
		plp,tres	4	0	–	–
		plim,tres	0	12	–	–
Does not solve	T-pattern detection	plim,dos	0	1	–	–
	Sequential analysis	nrs	–	–	-	32
		coj	–	–	12	–
		cosj	–	–	72	
		inc	–	–	394	
		plp,uno	12	0	–	–
		plp,dos	8	0	–	–
		plp,tres	24	0	–	–
		plim, dos	48	0	–	–

Table 10 shows how the lag sequential analysis detects a greater number of patterns and also more varied typologies (prospective/retrospective, complete/incomplete) for both groups of participants. In addition, with this data analysis technique more categories generate patterns, comparatively to T-pattern detection since the latter technique only allows to detect behavior patterns that occur in a time interval. Therefore, T-pattern detection in our study only allows to detect patterns about planning. Comparing the patterns generated from these categories, it is striking that precise planning (*plp*) generates patterns with any piece when lag sequential analysis is used, but not when using T-pattern detection, which only does it with precise planning for piece one (*plp,uno*). On the other hand, when dealing with imprecise planning (*plim*), lag sequential analysis generates patterns only with piece three (*plim,tres*) while T-pattern detection also finds patterns for piece three in addition to piece two (*plim,dos*).

Despite detecting differences in the results obtained with each data analysis technique, common aspects among them have also been found. Table 11 includes these aspects: the common aspects between the three techniques are highlighted and common aspects between two of them are underlined (T pattern-detection and polar coordinate analysis). No similarities were detected that only affect lag sequential analysis and polar coordinate analysis.

**Table 11:** Assessment of children metacognitive skills from the complementarity of the three data analysis techniques.

Participant group	Lag sequential analysis	T_Patters detection	Polar coordinate analysis
Solves	**plp,uno** plp,dos **plim,tres acpla,uno** nace,uno acpla,dos acpla,tres nrs coj taa,tres **rs**	**( plp,uno ((( plim,tres acpla,uno** )( taa,dos **rs** )) adin ))	
	**plp,uno** plim,dos **plim,tres acpla,uno** nace,uno acpla,dos tae,uno nrs coj **taa,tres rs**	(( **plp,uno plim,tres**)(( **acpla,uno taa,tres** ) **rs** ))	
	**plp,uno** plp,dos plim,tres **acpla,uno** nace,uno nace,dos acpla,tres nrs coj inc **rs**	(( **plp,uno** (**acpla,uno rs**)) coj )	
	**plp,uno** plp,dos plim,tres acpla,uno nace,uno **acpla,dos** tae,uno nrs coj tae,tres **rs**	( **plp,uno** (**acpla,dos rs**))	
	**plp,uno** plim,dos nace, tres acpla,uno nace,uno nace,dos **acpla,tres** nrs coj tae,tres **rs**	( **plp,uno** (**acpla,tres rs**))	
	**plim,tres acpla,uno** acple,dos acple,tres tae,tres **taa,dos** tae,tres **rs**	((( plim,tres **acpla,uno**)(**taa,dos rs**))(adin cosj))	plim,tres - adin // **plim,tres - taa,dos**
	**plim,tres** acple,dos **acpla,uno** acple,tres tae,tres tae,dos **taa,tres rs**	( **plim**,tres ( **acpla,uno** (**taa,tres rs**)))	**plim,tres - taa,tres**
	**plim,tres** plim,uno **acpla,uno** acple,tres tae,tres taa,dos taa,tres **rs**	(( **plim,tres acpla,uno**)(**rs** coj))	
Does not solve	**plim,dos** plim,tres acple,dos acple,tres acple,dos naca,tres **nrs** inc	((( **plim,dos** nrs )( tae,tres aday ))(( tae,dos aday ) **nrs** ))	

*Bold values indicate common aspects between the three data analysis techniques*.

## Discussion

The results obtained in this research from the mixed methods perspective represent a very important advance in the field of: (1) children metacognitive skills and (2) their assessment.

(1) Concerning children metacognitive skills, this study provides evidence on: (a) the ability to use these skills in preschool children, which corroborate results obtained by other researchers (Whitebread et al., 2009; Marulis et al., 2016; Roebers, 2017; Mari and Saka, 2018). More specifically, the study has allowed to know the sequential and associative structure of metacognitive skills that 5 year old children put into action during the resolution of a puzzle. These results highlight, once again, the importance of conducting studies in the natural context of children and using meaningful play tasks for them; (b) the existence of differences in these capacities between those participants who solved the puzzle and those who did not. We hypothesized that there would be differences both in their planning and in their monitoring and evaluation. However, the differences between the both groups of participants mainly affect their monitoring. The participants who solved the task, in the case of committing an error, that is, in the case of incorrectly solving the puzzle in a first attempt (*nrs*), were able to make their plan of action more flexible and make a new action with the corresponding piece, taking them to solve the puzzle (*rs*) (see Table 5 for patterns generated by *plp,uno*). However, the participants who did not solve the task showed a greater number of sequential structures in which incorrect resolutions appeared on their first attempt (see *nrs* in Table 7 for patterns generated by *plp,uno*; *plp,dos*; *plp,tres*, and *plim,dos*). After committing these errors they were not able to generate alternative actions to solve the task (see Table 7 for the patterns we have just indicated). This could be due to failures in their executive function of cognitive flexibility. Several studies defend the existence of relationships between metacognition and executive functions, although the mechanisms that would explain these relationships are still unknown (Bryce et al., 2015; Nelson and Marulis, 2017; Roebers, 2017).

As already mentioned, we expected to find differences in planning between children who solved the puzzle and those who did not, but this was not the case. Both groups of participants made accurate plannings (*plp*). Moreover, in both groups of participants the accurate planning of each of the three pieces of the puzzle (*plp,uno; plp,dos; plp,tres*) generated patterns (see Table 5 for participants who solved the puzzle and Table 7 for those that did not). However, these results imply important implications for the teacher's daily practice because they show that the mere fact of initiating the task correctly does not ensure its success (see Table 7 for patterns generated by *plp,dos* where it can be seen that despite precisely and sequentially planning the three puzzle pieces -*plp,dos; plp,tres; plp,uno*-, participants made mistakes in their execution and did not solve the task -*nrs*-). So, teachers must attend all the phases of execution of a child's task, and not merely its beginning.

The metacognitive difficulties detected already at these early ages may increase as children develop, with the repercussions that this implies since what happens in the first years of life is the basis for later learning (Scharf et al., 2016). This shows the need to address these difficulties as soon as possible and offer interventions tailored to the child's level, so that the child can benefit from the help offered. The teacher must be a guide and an aid in child learning (Robson, 2015; Perry et al., 2018). Consequently, the teacher must know the level of metacognitive skills of each of his/her students and how its sequentiality works to offer proposals that are within the area of the child's next development, in accordance with Vygotsky's concepts of scaffolding and the zone of proximal development (Vygotsky, 1978). If this is not the case, it is very difficult for the teacher's educational work to produce the desired effects (Perry et al., 2018). This is the situation that is detected in the group of children who did not solve the puzzle: despite interventions and assistance from adults (*aday*), children do not get to benefit from these proposals or interventions and therefore continue to produce errors in the placement of the pieces, without moving toward a successful resolution of the puzzle.

Although the Preschool Education curriculum in Spain (Education and Science Ministry of Spanish Government, 2008) makes multiple references to the “learning to learn” competence and the ability of learning and autonomous activity of children (highlighting the need for children to be more capable of making decisions, solving problems or using cognitive resources in an increasingly complex and elaborated way), it is true that in educational practice, in general, teachers devote little time to it. Usually, in the classroom, more instrumental learning is done, such as the beginning of basic numerical skills and reading and writing skills. In addition, for children to acquire these instrumental learnings it often happens that the teacher offers tasks too structured with rigid instructions that must be fulfilled by all children, not allowing the child to stop and think to make their own decisions on how to solve the task. We consider that it is necessary to allocate time for the student to think and explore different ways of solving the tasks, so that the professorate promotes the importance of the process and pays less attention to the quality of the result. In short, it is necessary that the educational environment promotes moments of constant reflection, before, during and at the end of all activity, so that children acquire the habit of stopping, thinking and acting on what they do. For this, it is appropriate for the teacher to act as a model and to accompany all of his/her actions with verbalizations that express and justify the different metacognitive skills that he/she is using to solve the task. It is important that the teacher models various strategic approaches to tasks, exemplifying a flexible use of procedures. Through modeling students can observe the processes that are required to perform the task, helping them to develop their metacognitive skills and their conscious use. Subsequently, the teacher must complete the modeling with metacognitive dialogues (also called posing questions; interrogation; questioning; mayeutics or socratic method of teaching). In these dialogues, the teacher raises questions about the process followed when acting (“*How did you do it?*”, “*Why did you do it that way?*”, “*Why did you say this?*”, “*Can you do it in a different way?*”, etc.), so that the child must think and reflect on it. Through these dialogues and questions adults offer strategy models of self-questioning, self-diagnosis, and self-correction, transferring to the child the control and planning of their own activity. Thus, the objective of the technique is to ensure that students become aware of their own thought processes. Although this will not be achieved until later ages -given that even in adolescence metacognitive skills continue to improve (Roebers, 2017)—it is necessary to work in this direction from an early age; but always with enjoyable tasks, as has been done in this study.

(2) With respect to the assessment of children metacognitive skills, the realization of this study entails contributions for: (a) the practical and applied evaluation of these skills in the classroom of Preschool Education by the teacher. The Spanish educational legislation requires that in Preschool Education the teacher assesses the learning and development of students through direct and systematic observation (Education and Science Ministry of Spanish Government, 2008). However, it is not always done that way. Often, observation is used inaccurately in schools, without clear criteria and in a non-objective way, in addition to focusing only on the results or final product and not on the process followed by the child. This means that sometimes information may be lost about the progress the child is making in the process (although still not achieving a successful result) and therefore, obtain information that reflects a false stagnation or involution in his/her development and learning. This study helps to avoid these malpractices since an observation instrument is offered (see Table 1) whose use allows the teacher to evaluate the metacognitive skills of his/her students in an objective and rigorous manner, focusing on their process as a result and obtaining reliable information. This allows to offer a sensitive educational response tailored to the needs of students. In short, the built observation instrument facilitates the teacher's assessment of their students and contributes to the objectivity of the same, with the benefits that this entails for children; (b) This study also entails methodological contributions for the evaluation of children metacognitive skills in the field of research.

The complementary use of three powerful data analysis techniques allows a rigorous, objective, and exhaustive assessment. It implies a methodological advance in the research field. Therefore, in general terms, it can be said that the combined use of both techniques has provided more exhaustive information about the sequentiality of the use of metacognitive skills in participants than if only one analysis technique had been used. Now, focusing on the results offered by each of them individually, in this study the lag sequential analysis is the one that has provided the most information. This fact could initially seem contradictory to what was found by other authors (Santoyo et al., 2017; Tarragó et al., 2017), who affirm that T-pattern detection offered in their researchs a more exhaustive and profound information. However, it should be noted that in our study lag sequential analysis was performed considering 10 prospective lags and 10 retrospective ones, while in other studies (such as those cited), only five prospective and five retrospective lags are calculated. It is true that the consideration of five delays both prospectively and retrospectively is a usual practice with this type of analysis, although in the present study given the subject, its aim and participant characteristics it would have been insufficient to do so and therefore, information would have been lost. It would be interesting that in future studies this usual practice of considering five delays on lag sequential analysis is extended to 10 and that results obtained are also compared with those found with the use of T pattern-detection.

Until now, studies focusing on this type of data analysis techniques have mainly used these techniques in isolation, without seeking complementarity (Arias-Pujol and Anguera, 2017; García-Fariña et al., 2018; Maneiro and Amatria, 2018). There are recent works that have begun to use them in addition, but it has been mainly two to two (Castañer et al., 2017). We only know two papers (Santoyo et al., 2017; Tarragó et al., 2017) that have addressed the complementarity of the three techniques, and although one of them has been developed in the educational context, it has been used with more senior students and analyzing activity organization in the classroom.

In conclusion, the complementary use of these three techniques of observational data analysis is an important contribution to the field of metacognitive skills for children and their assessment, and therefore also to the field of learning and early childhood education, both at the practical and research levels.

These numerous contributions and implications of the study could be even greater if the following limitations could have been overcome: observe the implementation of the metacognitive skills of children in a single moment of the course and in a single playful task.

In order to overcome these limitations and keep moving forward in the field, in the future it would be interesting to: (a) track the participants to know the development of their metacognitive skills in the following school years. This would make a great contribution to this field of study because there are hardly any longitudinal studies on the development of metacognitive skills, and even less at early ages (Paulus et al., 2013). We also consider that this would be of great interest, taking into account that in the next course these participants will begin Compulsory Education, characterized by more complex tasks and higher academic demands; (b) to estimate the predictive power of metacognitive skills for academic achievement but not only in mathematics and literacy but also in different components of them (mental calculation, solving mathematical problems, reading comprehension, etc.). If preschool metacognitive skills turned out to be good predictors of these academic competences, it would be possible to detect children who are likely to present different academic difficulties later and intervene preventively according to their limitations, contributing in this way to their academic success; (c) compare the metacognitive skills of children in cooperative learning tasks with those demonstrated in individual tasks, since several studies indicate that social interactions can facilitate the development of metacognitive skills (Chatzipanteli et al., 2014); (d) collect information on the teaching style of the teaching staff, since it is one of the variables that most affects the development of the children's metacognitive skills, but has been scarcely analyzed (Roebers, 2017).

## Ethics Statement

This study was part of a wider research which was evaluated and approved by the Research Unit of Zaragoza University. Research was also approved by the school management team and teachers. In accordance with the Organic Law 15/1999 of December of Protection of Personal Data (1999, BOE n_ 298 of December 14) all the parents of the participants signed the informed consent authorizing the participation of their son/daughter in the study and being recorded while playing. In addition, and following the guidelines of the aforementioned law, the observers signed the confidentiality agreement. No ethics special approval was required for this research since the Spanish public education system and national regulations require no such approval. Each participant received a small reward (two chocolates) in gratitude for their participation.

## Author Contributions

EE-P contributed to conceptual structure, collecting data, systematic observation and performed data analysis and results. She was responsible for the literature review and the drafting of this manuscript. MH-N involved in collecting data, systematic observation and performed data analysis and results. MTA contributed to methodological structure, offered guidance on data analysis, performed and supervised them. All of the authors contributed to revising the manuscript and provided final approval of the version to be published.

### Conflict of Interest Statement

The authors declare that the research was conducted in the absence of any commercial or financial relationships that could be construed as a potential conflict of interest.
